# Treatment of Fractured Legs

**Published:** 1890-08-16

**Authors:** 


### TREATMENT OF FRACTURED LEGS.

In a recent number of the *Lancet* Mr. G. R. Foulerton, assistant surgeon to St. Bartholomew's Hospital, Chatham, gives some notes on a method of making plaster splints, more especially for the treatment of fractures of the leg, and more particularly for such injuries as are suitable for "immediate" treatment. In the case of a fracture, say at the junction of the middle and lowest thirds of the limb, three measurements are taken: 1. The length of the leg from an inch and a-half above the knee to the sole of the foot. 2. The circumference of the thigh at the heighth of about an inch and a-half above the knee. 3. The length of the foot from the heel to the ball of the big toe. An oblong piece of shrunk house-flannel, or old blanket, is then cut out, of such dimensions that it is in one way an inch and a-quarter longer than measurement; the other way, and inch and a-half less than twice the measurement of 2. The limb is laid on the piece of flannel, which is then held up by its edges, supporting the leg as in a sling. Next the flannel is tacked together with needle and thread close to the limb, down the front of the leg, along the sole, and then along the dorsum of the foot. The leg is thus closely encased in flannel, whilst along the front of the leg and dorsum and sole of the foot, a double free edge of flannel of greater or less extent is left beyond the seam. These free edges along the dorsum of the foot as high as the ankle, and along the sole, are trimmed off close to the seam. There now remain two considerable wings of flannel extending down the front of the leg as far as the ankle. Each wing, when turned back from the middle line in front, will reach to within about half-an-inch of the middle line behind. These wings are then trimmed from below upwards, so as to shape them to the form of the leg. Two side splints with rectangular foot-pieces are then cut out of flannel, similar in shape to those used in a "Croft." Each piece, when applied to the limb, should reach to within about a quarter of an inch of the middle lines in front, and behind. Traction is then applied to the limb, and these side pieces having been thoroughly saturated with plaster-of-Paris solution, are placed over the flannel casing, one on each side of the limb. The wings of flannel are then quickly folded back, each over its respective side piece, and smoothed down. Plaster solution is then rubbed well into the outer border of the two flannel wings, avoiding, however, a margin about a quarter of an inch wide, and extending down the front of the limb on either side of the seam. The perfect adaptation of the splint to the limb may be secured by applying a thin muslin roller over the whole from the toes upwards. After ten minutes the muslin bandage is unrolled, and the plaster will be quite dry in an hour or so. When the splint is thus completed, there is next to the skin a continuous casing of flannel. Outside this there is on either side, except just below the ankle, a double thickness of plaster-saturated flannel. Along the middle, behind, there is a sort of hinge, formed by the single thickness of plaster-free flannel of the original casing, and each anterior edge is formed by a fold of soft flannel. This latter is of importance as the skin is thereby protected from uncomfortable pressure of the anterior edges of the plaster saturated side pieces. With these splints there is no tendency to the formation of superficial bullæ from such cause; nor will subsequent padding with cotton wool be found necessary. After the splint is quite dry it can be easily opened by running the scissors down the seam along the front of the leg, and dorsum of the foot, and the splint may be afterwards held together by an ordinary roller. The author states this splint is more durable and stronger than any other, and he advises its use when a splint has to be opened more or less frequently, as well as in cases where immediate cure is the aim.

A very similar procedure is given in Stanley Boyd's edition of Druit's "Surgeons' Vade Mecum." "Cut two pieces of coarse flannel, each like two Cline's splints joined together, and large enough to surround the leg and foot, with two inches to spare. Fasten them together with two rows of stitching half an inch apart along the mid-line behind. Place them beneath the leg, and bring together the inner by stitches along the front of the leg, dorsum, and sole of the foot. Thick plaster cream is now rubbed in and spread smoothly over to the depth of half an inch; then the outer pieces are brought together firmly, pressed down on the plaster, and cut off close to the mid-line. When necessary, the stitches in front may be cut and the splint opened, the seam along the back acting as a hinge. It is re-applied with a bandage." In respect of a special flannel casing for the limb, Mr. Foulerton's process may be an improvement. We are by no means satisfied, however, that treatment as above is the most desirable method. If there is no eversion of the foot two lateral hollow splints may be used, one reaching from the knee to the extremity of the toes, the internal extending as far as the tarsus. The limb may also be supported in a cradle. At the end of three weeks a starch bandage. When there is eversion, a Dupuytren's splint to the inner side of the leg will often prove satisfactory.

